# Epidemiology of dengue fever in Gabon: Results from a health facility-based fever surveillance in Lambaréné and its surroundings

**DOI:** 10.1371/journal.pntd.0008861

**Published:** 2021-02-10

**Authors:** Jacqueline Kyungah Lim, José Francisco Fernandes, In-Kyu Yoon, Jung-Seok Lee, Regis Obiang Mba, Kang Sung Lee, Suk Namkung, Jae Seung Yang, So Hee Bae, Sl-Ki Lim, Bertrand Lell, Meral Esen, Marguerite Massinga Loembe, Peter G. Kremsner, Neal Alexander, Selidji Todagbe Agnandji

**Affiliations:** 1 International Vaccine Institute, Seoul, Republic of Korea; 2 Centre de Recherches Médicales de Lambaréné, Campus CERMEL, Lambaréné, Gabon; 3 Department of Medicine I, Division of Infectious Diseases and Tropical Medicine, Medical University of Vienna, Vienna, Austria; 4 Institute of Tropical Medicine, University of Tübingen, Tübingen, Germany; 5 Coalition for Epidemic Preparedness Innovations (CEPI), Washington, DC, USA; 6 German Centre for Infection Research (DZIF), Partner Site Tübingen, Germany and CERMEL, Lambaréné, Gabon; 7 MRC International Statistics and Epidemiology Group, London School of Hygiene and Tropical Medicine, London, United Kingdom; Centre hospitalier de Cayenne, FRANCE

## Abstract

**Background:**

In Africa, information on dengue is limited to outbreak reports and focused on some countries with continuing transmission in West and East Africa. To estimate the proportion of dengue-positive cases among febrile patients and identify clinical indicators of dengue cases, we conducted passive facility-based fever surveillance in a catchment area population of 70,000 residents of Lambaréné and its surroundings in Gabon.

**Methods:**

Non-malarial febrile patients with current fever or history of fever (≤7 days) between 1 and 55 years of age, were enrolled at Albert Schweitzer Hospital (ASH). Acute (visit 1, day of enrollment) and convalescent blood samples were collected between 10 and 21 days after enrollment. Acute/convalescent samples were tested with IgM/IgG ELISA, and a selected subset of acute samples with RT-PCR.

**Results:**

Among 682 non-malarial febrile patients enrolled, 119 (17.4%) were identified as dengue-positive (94 dengue-confirmed and 25 dengue-probable cases). Of these dengue-positive cases, 14 were confirmed with PCR, and based on serotyping, two infections were identified to be DENV-2 and two were DENV-3. The majority of our enrolled patients were <25 years of age and close to 80% of our dengue-positive cases were <15 years of age. In adjusted analyses, retro-orbital pain and abdominal pain were 2.7 and 1.6 times more frequently found among dengue-positive cases, compared to non-dengue cases.

**Conclusion:**

Lambaréné is not considered dengue-endemic. However, one in six non-malarial febrile episodes was found to be dengue-positive in the study period. Dengue should be considered more frequently in clinicians’ diagnosis among non-malarial febrile patients in Lambaréné. Given the lack of data on dengue in Gabon, additional prospective and longitudinal studies would help to further define the burden and patterns of dengue for improved case detection.

## Author summary

In Africa, information on dengue is limited to outbreak reports focused on some countries in West and East Africa. To estimate the proportion of dengue-positive cases among febrile patients and identify clinical indicators of dengue cases, we conducted passive health facility-based fever surveillance in a catchment area population of 70,000 residents of Lambaréné and its surroundings, Gabon. Among the patients with negative malaria RDT results, those with current fever or history of fever (≤7 days) between 1 and 55 years of age were enrolled at Albert Schweitzer Hospital (ASH). Two samples were collected with an interval of 10 to 21 days after enrollment. Samples underwent different testing for dengue confirmation. Among 682 febrile patients enrolled, 17.4% were identified as dengue-positive. Of these dengue-positive cases, we found DENV-2 and DENV-3 serotypes. Close to 80% of our dengue-positive cases were < 15 years old. Retro-orbital pain and abdominal pain were more commonly found among dengue-positive cases, compared to non-dengue cases. Lambaréné is not considered dengue-endemic. However, one in six non-malarial febrile episodes was found to be dengue-positive in the study period. Clinicians should consider dengue more frequently among non-malarial febrile patients. Given the lack of data on dengue in Gabon, more data should be generated to understand the burden and patterns of dengue for improved case detection.

## Introduction

Dengue fever (DF) is a mosquito-borne flavivirus infection caused by four related but antigenically distinct dengue viruses (DENVs, serotypes 1–4). As a major and rapidly increasing global public health problem, there are approximately 50 to 100 million cases of DF and 500,000 severe dengue cases requiring hospitalization reported annually worldwide [1–3].

With the known presence of the *Aedes* mosquito vectors in Africa, dengue cases have been reported in 34 African countries [4–6]. However, most are from the same few countries in the region, in particular from outbreak investigations [5,7,8]. Recently, DENVs have been identified as a common cause of febrile illness in Africa, but there are continued challenges in terms of diagnostic capabilities limiting accurate estimation of the burden among many causes of febrile illness [9–11].

The burden of dengue remains largely unknown in Gabon, although a recent study demonstrated continued circulation of DENV-3 in Lambaréné [12]. Another study from Lambaréné showed 12.3% sero-prevalence using a commercial enzyme-linked immunosorbent assay (ELISA) for dengue IgG-antibodies among infants at 30 months of age [13]. On the other hand, an earlier study in 2005–2008 in a random selection of about 10% of all Gabonese villages detected minimal levels of IgG and IgM positivity against dengue [14–16]. Furthermore, there is one study claiming the absence of dengue virus circulation in the rural part of Gabon [17].

Most African countries lack mandatory reporting or national surveillance systems for dengue [18]. Dengue is not a notifiable disease in Gabon nor is there a routine national monitoring. To better understand the dengue situation and characterize dengue epidemiology in Gabon, a passive health facility-based fever surveillance study was conducted in Lambaréné and its surroundings from May 2015 to December 2016.

## Methods

### Ethics statement

The study protocol received ethical approvals from the Institutional Review Boards (IRBs) of IVI (#2019–003), the London School of Hygiene and Tropical Medicine (#8794), Gabon National Ethics Committee and Institutional Ethics Committee (No. 007/2015/SG/P) of CERMEL in Gabon. A written informed consent form (ICF) was obtained from each participant. For those aged between 11 and 17 years, an assent form was obtained, plus informed consent from at least one parent or legal guardian.

### Study area and population

Gabon is on the west coast of Central Africa. Approximately 87% of the population live in urban areas [19], with Lambaréné being the sixth largest city. Capital of Moyen-Ogooué province, Lambaréné is located 75 kilometers south of the equator, with a population of 38,775 in 2013 [20]. The population of Lambaréné is relatively young with more than 50% under 20 years of age [21].

Site selection took into account existing evidence on DENV transmission and adequate research infrastructure to implement the studies [4,22]. The study was launched in May 2015 in Lambaréné and its surroundings in Moyen-Ogooué province, a semi-urban area, in collaboration with the Centre de Recherches Médicales de Lambaréné (CERMEL).

**Fig 1:**
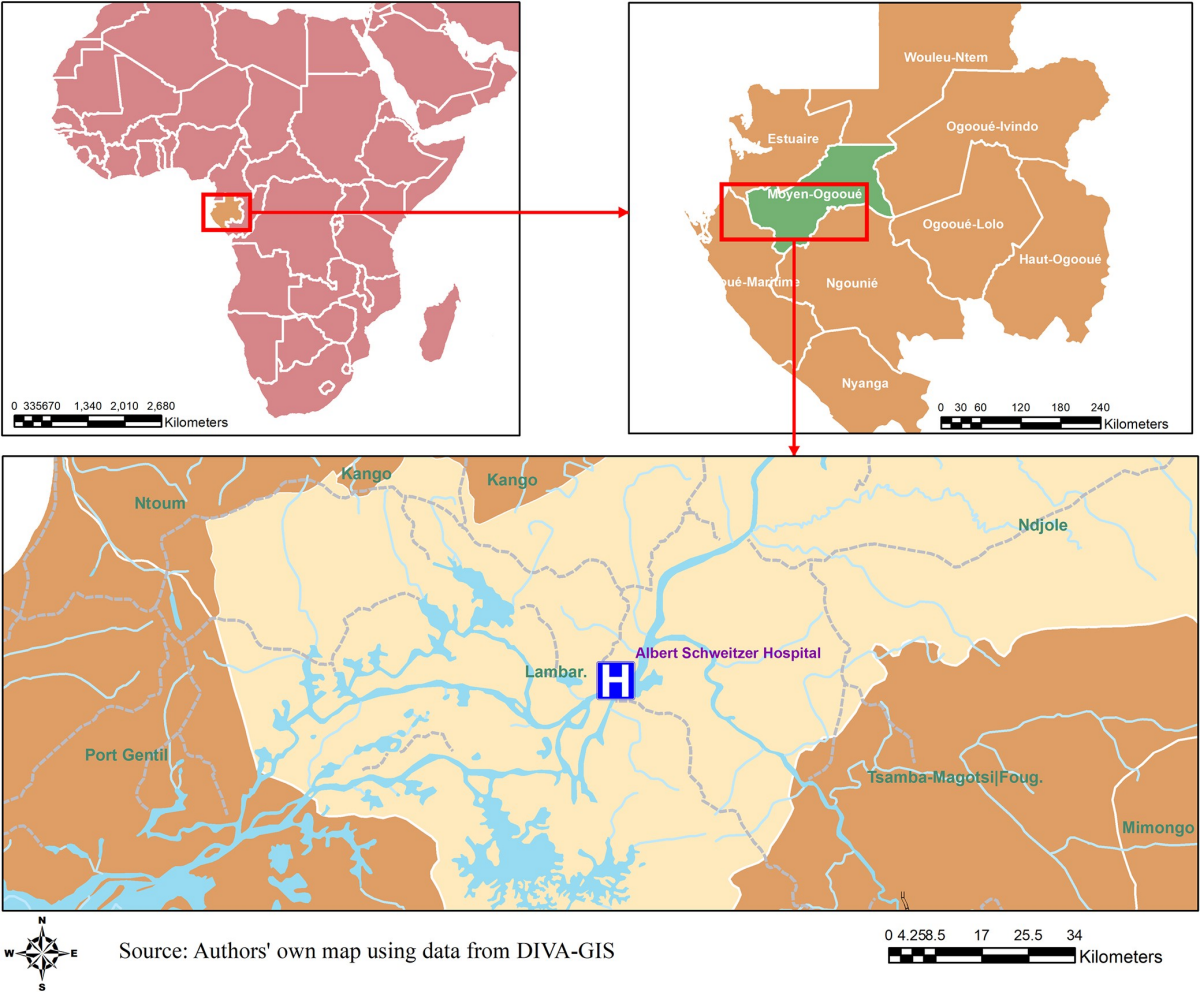
Map of the study area. A map of the study area in Lambaréné and its surroundings, Gabon.

The city’s health care infrastructure consists of two primary health centers, 10 dispensaries, and two main hospitals, the Albert Schweitzer Hospital (ASH) and the Lambaréné General Hospital (GH), each of which is equipped with a pediatric ward, surgery, internal medicine, antenatal clinics and emergency services (open 24 hours). ASH is a public-private institution which is the main hospital providing care for the catchment area population of approximately 70,000 residents [23,24]. Fig 1 shows the area of Lambaréné and its surroundings, the catchment area for the study in Gabon.

### Study design

The study enrolled both outpatients and inpatients at ASH between May 2015 and December 2016 (20 months). As described in a published protocol [22], patients presenting with fever (body temperature ≥ 37.5° C) or history of (i.e. self-reported) fever for ≤7 days were tested for malaria using thick blood smear as part of routine practice. Patients were eligible for study enrolment if they were malaria negative, without localizing signs (i.e., no localized infection or known/confirmed non-dengue etiology), aged 1–55 years, resident in the hospital’s catchment area, and provided informed consent, plus assent for individuals aged 11–17 years. Also, infants < 1 year old were not included due to operational limitations, such as difficulty of venipuncture.

An acute sample of blood (7–10 ml) was taken when the patient first visited the hospital with (current or history of) fever. Then, a study physician/nurse conducted interviews and physical exams, and a case report form was completed capturing symptom history, medical history, treatment, and laboratory results [22]. A convalescent blood sample was collected at the facility between 10–14 days after the initial visit, or if not possible within this timeframe, a home visit was made within 21 days.

### Laboratory testing algorithm

Acute and convalescent sera were tested at the CERMEL laboratory using dengue IgM/IgG ELISA (SD Dengue IgM & IgG Capture ELISA, Standard Diagnostics, Yongin-Si, Korea)[22]. Furthermore, Reverse Transcription Polymerase Chain Reaction (RT-PCR) was performed at International Vaccine Institute (IVI) on acute sera from patients who had [25]: sero-conversion between acute and convalescent samples by IgM and IgG capture ELISA. RT-PCR was also performed on a limited number of randomly selected acute sera that were: sero-positive in both acute and convalescent samples by IgM and IgG capture ELISA; or negative by ELISA on all samples. In addition, a subset of these samples was tested at IVI using a commercial Bioneer triplex real-time RT-PCR kit for dengue virus (DENV), Zika virus (ZIKV), and chikungunya virus (CHIKV) detection. For these results, serotyping information was not provided.

Dengue infection status was categorized based on the interpretation of laboratory results, following the WHO diagnostic criteria [26]. Sero-conversion by dengue IgM and/or IgG between acute and convalescent samples and/or virus detection by RT-PCR in the acute sample were considered to be laboratory-confirmed dengue. Positive IgM by ELISA in a single acute sample or paired acute/convalescent samples were considered as probable dengue [26]. Dengue-confirmed and -probable cases were combined into a dengue-positive group for this analysis. Patients with negative RT-PCR and negative paired acute/convalescent IgM ELISA were classified as non-dengue.

### Statistical analysis

There were two components in the analysis. First, a descriptive summary of clinical and laboratory characteristics is presented for dengue-positive and non-dengue cases. To indicate elevated body temperature among the enrolled febrile patients, as a dichotomous variable, was defined as body temperature ≥38.5°C, the 75th percentile of the body temperature measured at enrollment. Clinical diagnosis (i.e., made by a clinician prior to laboratory confirmation) was grouped as suspected dengue, undifferentiated fever, and other illness. Yellow fever (YF) vaccination history was dichotomized between those who reported having been vaccinated versus those who did not remember or reported no vaccination. Complete blood count (CBC) was generally only done for hospitalized patients and were missing for outpatients, so these results were not included in the analysis.

Secondly, to assess how clinical presentation of febrile cases differed by dengue infection status, logistic regression was used to build a multivariable model of clinical indicators. The model contained age and gender as a priori confounders, being possibly associated with exposure to *Aedes* vectors, and with some clinical features [27]. Also, despite lack of immunological foundation in association of YF vaccination and dengue infection, there has been a report on predisposition of YF vaccinated individuals to a severe form of dengue [28]. Thus, having received YF vaccination was also included as a priori confounder.

A backward stepwise process was used to select a final multivariable model, with a significance level of 0.2 for entry and 0.1 for retention. Further variables investigated included: demographic and clinical variables such as inpatient/outpatient, the duration of fever prior to enrollment, the temperature at presentation, and clinical signs/symptoms. Some signs and symptoms were used only in the descriptive and univariate analyses, due to data sparsity. Clinical diagnosis of suspected dengue was not included because a) it was considered to be closely related to dengue infection status and b) since the intended output is to inform clinical decision-making, it would not be logical to include clinical diagnosis as an input.

As part of sensitivity analysis, a descriptive summary of clinical and laboratory characteristics and univariable analyses using three (rather than two) categories for dengue infection status—confirmed, probable, and non-dengue—is presented in supplementary information (S1 Table).

Categorical pair-wise comparisons were made across dengue infection status using χ^2^ or Fisher’s exact tests with a significance level of 0.05. Continuous variables were compared using Student’s *t*-test or ANOVA. All analyses were performed using SAS version 9.4 (SAS Institute, Cary, North Carolina).

## Results

**Fig 2:**
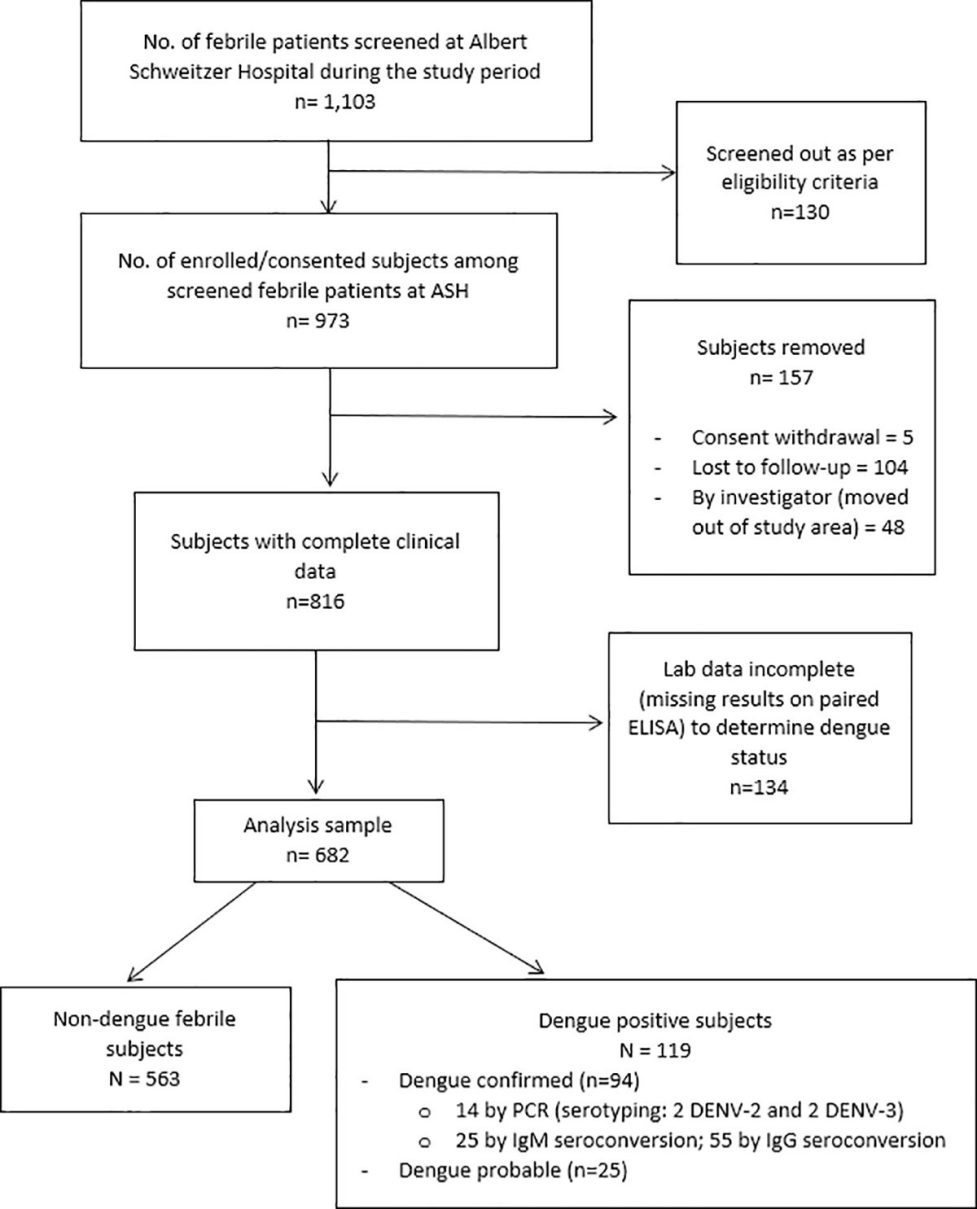
Flow chart describing the ascertainment of the febrile patients. The chart describes patient flow in the passive fever surveillance from screening, enrollment to study participation, with determination of laboratory-based status of dengue infection, as well as how the analysis sample was reached.

Of 973 enrolled patients, 157 did not complete the study, and the laboratory data of 134 others were insufficient for determination of dengue infection status (i.e. lacking paired ELISA results), leaving 682 for the main analysis (Fig 2). Although similar in terms of gender, the 134 patients with incomplete laboratory data were significantly older (mean age = 19.5 years), compared to those in the analysis sample (mean 9.1 years), and had slightly more days of illness before enrollment (mean 3.9 days, compared to 3.2 days in the analysis sample).

### Clinical characteristics between dengue-positive and non-dengue cases

**Fig 3:**
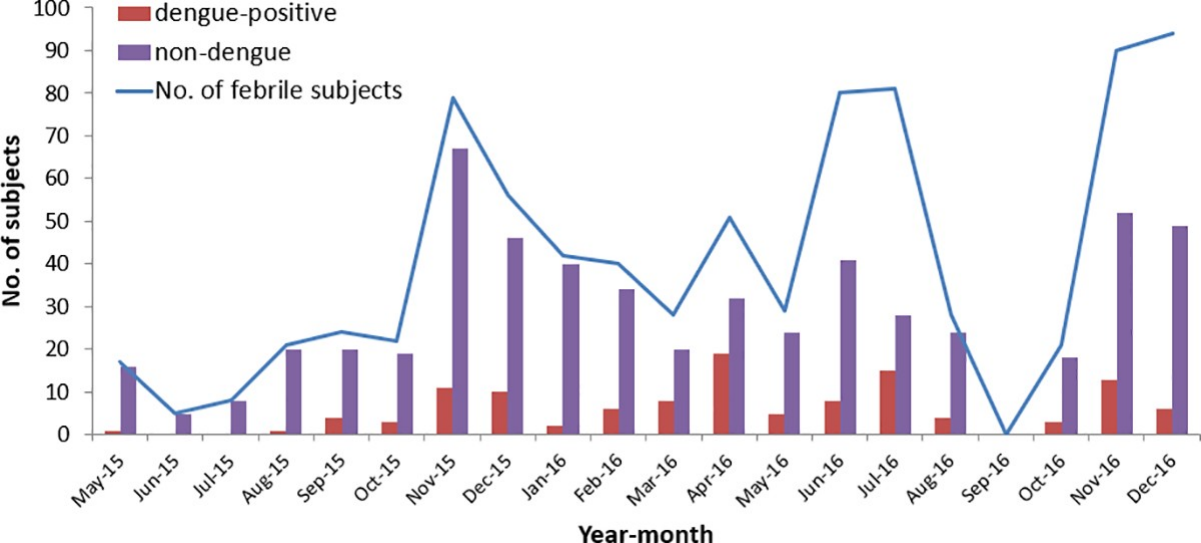
Monthly distribution of the enrolled febrile patients and dengue-positive patients. The figure shows monthly distribution of dengue-positive and non-dengue cases among the enrolled patients.

Table 1 describes demographic and clinical characteristics of dengue infection status (positive vs. non-dengue). The three-level classification (dengue-confirmed, probable, and non-dengue) is presented in the supplementary table (S1 Table). Of 682 patients in the analysis sample, 563 (82.6%) were non-dengue and 119 (17.4%) were dengue-positive (Fig 2). Of the 119 dengue-positive patients, 94 were laboratory-confirmed and 25 were probable dengue. Of the 94 dengue-confirmed cases, 14 (14.9%) were confirmed by PCR; 25 (26.6%) by seroconversion on IgM ELISA between acute and convalescent samples, and the remaining majority (n = 55) by seroconversion on IgG ELISA (Fig 2). Small peaks in dengue-positive cases were observed in October-December and February-May (Fig 3). Based on PCR serotyping information, DENV-2 and DENV-3 circulated during our study period. Also, using a commercial triplex (DENV, CHIKV, and ZIKV) kit, we retrospectively tested a subset of samples (n = 62) and none was found to be chikungunya- or Zika-positive.

**Table 1:** Demographic and other baseline characteristics of the dengue infection status (dengue-positive and non-dengue) among patients with non-malarial febrile illness identified in the health facility-based fever surveillance established in Lambaréné, Gabon in 2015–2016.

Characteristics	Total (n = 682)	Dengue-positive (n = 119)	Non-dengue (n = 563)	p-value
Mean age (SD)	9.15 (7.66)	8.96 (6.57)	9.19 (7.88)	0.766
Age group (years)				0.698
1–4	233 (34.2)	40 (33.6)	193 (34.3)	
5–9	203 (29.8)	35 (29.4)	168 (29.8)	
10–14	105 (15.4)	19 (16.0)	86 (15.3)	
15–19	83 (12.2)	13 (10.9)	70 (12.4)	
20–24	43 (6.3)	11 (9.2)	32 (5.7)	
25–34	4 (0.6)	1 (0.8)	3 (0.5)	
35–55	11 (1.6)	0	11 (2.0)	
Fever duration prior to visit				0.080
1–2 days	226 (33.1)	29 (24.4)	197 (35.0)	
3 days	228 (33.4)	46 (38.7)	182 (32.3)	
4–7 days	228 (33.4)	44 (37.0)	184 (32.7)	
Fever duration, entire illness	3.22 (1.24)	3.41 (1.24)	3.18 (1.23)	0.059
Temperature at presentation (SD)	38.31 (0.81)	38.39 (0.89)	38.29 (0.80)	0.211
Temperature at enrollment				0.514
Below 38.5°C	509 (74.6)	86 (72.3)	423 (75.1)	
≥ 38.5°C	173 (25.4)	33 (27.7)	140 (24.9)	
Prev. dengue infection (self-report)	0	0	0	-
YF vaccination	473 (69.4)	85 (71.4)	388 (68.9)	0.589
Inpatients/Outpatients	116 (17.0)/566 (83.0)	24 (20.2)/95 (79.8)	92 (16.3)/471 (83.7)	0.313
Mean days of hospitalization among IPD patients (SD)	4.58 (1.35)	4.33 (1.74)	4.66 (1.21)	0.338
Female	321 (47.1)	49 (41.2)	272 (48.3)	0.157
Clinical diagnosis				
Suspected dengue	5 (0.7)	4 (3.4)	1 (0.2)	**< .001**
Non-dengue	677 (99.3)	115 (96.6)	562 (99.8)	
Viral syndrome (% of non-dengue)	247 (36.5)	40 (34.8)	207 (36.8)	
Malaria	236 (34.9)	44 (38.3)	192 (34.2)	
Diarrheal illness	99 (14.6)	15 (13.0)	84 (15.0)	
Bronchitis	39 (5.8)	4 (3.5)	35 (6.2)	
URI	13 (1.9)	2 (1.7)	11 (2.0)	
Otitis media	5 (0.7)	2 (1.7)	3 (0.5)	
Other	38 (5.6)	8 (7.0)	30 (5.3)	
Signs and symptoms (presence)				
Rash	30 (4.4)	6 (5.0)	24 (4.3)	0.707
Fatigue/weakness	248 (36.4)	53 (44.5)	195 (34.6)	**0.041**
Headache	277 (40.6)	51 (42.9)	226 (40.1)	0.584
Retro-orbital pain	25 (3.7)	9 (7.6)	16 (2.8)	**0.013**
Neck pain	4 (0.6)	2 (1.7)	2 (0.4)	0.143
Ear pain	7 (1.0)	2 (1.7)	5 (0.9)	0.436
Breathing difficulty	19 (2.8)	4 (3.4)	15 (2.7)	0.675
Nasal congestion	154 (22.6)	24 (20.2)	130 (23.1)	0.488
Rhinorrhea	119 (17.5)	16 (13.5)	103 (18.3)	0.205
Sore Throat	12 (1.8)	3 (2.5)	9 (1.6)	0.487
Cough	376 (55.1)	61 (51.3)	315 (56.0)	0.350
Sputum production	288 (42.2)	46 (38.7)	242 (43.0)	0.385
Nausea & vomiting	289 (42.4)	49 (41.2)	240 (42.6)	0.771
Diarrhea	222 (32.6)	35 (29.4)	187 (33.2)	0.421
Constipation	36 (5.3)	10 (8.4)	26 (4.6)	0.093
Abdominal pain	187 (27.4)	43 (36.1)	144 (25.6)	**0.019**
Petechiae	6 (0.9)	1 (0.8)	5 (0.9)	0.960
Loss of appetite	488 (71.6)	82 (68.9)	406 (72.1)	0.481
Muscle pain	152 (22.3)	28 (23.5)	124 (22.0)	0.720
Joint pain	154 (22.6)	28 (23.5)	126 (22.4)	0.785

°C: degree Celsius; IPD: Inpatient Department; OPD: Outpatient Department; SD: Standard Deviation; URI: Upper respiratory infection; YF: Yellow Fever.

Close to 80% of enrolled febrile patients were children under 15 years of age with no evidence of a difference in age distribution between dengue-positive and non-dengue cases (Table 1). About 17% of the enrolled subjects were hospitalized and there was no significant difference in the proportion of hospitalized patients or the mean duration of hospital stay between dengue-positive and non-dengue cases.

Dengue-positive patients tended to seek care later than non-dengue patients, but the difference was only marginally significant. Overall, more than 95% of the enrolled patients had clinical diagnosis other than dengue and, of 5 patients clinically diagnosed with suspected dengue, 4 were found to be dengue-positive. Among those with clinical diagnosis other than dengue, viral syndrome and malaria were common diagnoses. In terms of symptoms, fatigue, retro-orbital pain, and abdominal pain were found more commonly among dengue-positive cases, compared to non-dengue. Based on the univariate logistic analysis, these variables found to be associated with increased odds of dengue positivity, compared to non-dengue, are listed in the Supplementary information (S2 Table).

The multivariable model building process selected retro-orbital pain and abdominal pain. With age, gender, treatment type (IPD/OPD), having received YF vaccination, and higher body temperature at enrollment as a priori adjustments and the duration of fever prior to visit identified to be significantly associated in the variable screening/selection process, we performed the final model with retro-orbital pain and abdominal pain.

Table 2 shows that dengue-positive cases were associated with 2.7 times and 1.6 times greater odds of presenting with retro-orbital pain and abdominal pain, compared to non-dengue cases. Also, dengue-positive patients were 1.7 times more likely to seek care on day 3 into febrile illness, rather than earlier.

**Table 2:** Multivariable logistic analysis showing significant indicators and their odds ratios of dengue positivity in the health facility-based fever surveillance.

Characteristics	Multivariable analysis (n = 682) *ref*. no dengue (n = 563)		
	Dengue-positive (n = 119)		p-Value
	aOR	95% CI	
Female (*ref*. Male)	0.77	0.51–1.17	0.218
Age (years)			0.970
1–4	Ref		
5–9	0.97	0.58–1.62	
10–14	0.98	0.52–1.83	
15–19	0.94	0.46–1.89	
20–55	1.25	0.59–2.64	
IPD (*ref*. OPD)	1.06	0.62–1.81	0.830
Temperature at enrollment			0.679
Below 38.5°C	Ref		
≥ 38.5°C	1.10	0.70–1.75	
Fever duration prior to visit			0.087
1–2 days	Ref		
3 days	**1.72**	**1.02–2.88**	
4–7 days	1.65	0.98–2.79	
YF vaccination ^A^			0.756
Not received vaccination	Ref		
Received vaccination	1.08	0.67–1.74	
Presence of signs and symptoms (*ref*. absence)			
Retro-orbital pain	**2.67**	**1.12–6.37**	**0.027**
Abdominal pain	**1.61**	**1.03–2.54**	**0.038**

IPD: Inpatient Department; OPD: Outpatient Department; YF: Yellow Fever; aOR, adjusted odds ratio.

^A^based on self-report

## Discussion

Overall, evidence on dengue in Africa is limited, and the available data tend to be from a few specific countries [5]. In the absence of marked lack of data on dengue in the central part of Africa, our study showed that dengue is a common cause of non-malarial febrile illness in patients in Lambaréné, Gabon.

### General characteristics of dengue-positive cases

Approximately one in six non-malarial febrile episodes was identified as dengue-positive. There are not many comparable data from Gabon. Abe et al. recently demonstrated DENV-3 circulation in 2016–2017 in Lambaréné [12]. This facility-based surveillance study enrolled 1007 febrile patients and confirmed 17 DENV cases by qRT-PCR with all 17 being DENV-3 [12]. Based on phylogenetic analyses, this study confirmed DENV-3 in a stable circulation since 2010 in Gabon [12]. The rate of detection of 1.7% (n = 17/1007) by PCR by Abe et al. is comparable to our data where the detection rate is 2.1% (n = 14/682) just considering our 14 PCR-positive cases, covering an overlapping study period of 2015–2016 [12].

While the age distribution did not differ between dengue vs. non-dengue cases in our data, it was a clear limitation to have adults underrepresented in our analysis sample, with only <3% of enrolled subjects over 25 years of age. Our study enrolled patients between 1–55 years, and the mean age among dengue-positive cases was 9.0 years, 8.7 years for dengue-confirmed cases (S1 Table). In the study by Abe et al., which included almost all ages (1–82 years), DENV-positive patients were older, with the mean age of DENV-3 confirmed patients being 29 years, ranging from 4 to 57 years [12]. This was consistent with a finding from another study based on multiple sites across Gabon by Caron et al. on DENV-positive cases during 2007–2010 over epidemic and inter-epidemic periods [14]. Although this previous study only had a single case from Lambaréné (DENV-2) with the rest from other parts of Gabon, the mean age was much older, compared to our results: 32.5 years among 386 DENV-2 confirmed patients; and 29.2 years among 20 DENV-1 patients [14]. Despite differences in the study period and location, Caron et al. and Abe et al. reported similar patterns in terms of age among dengue-positive patients, although different from the current study. It may be speculated that there may be older age groups protected with pre-existing immunity against these serotypes from previous epidemics, leaving children and young adults to be susceptible in the current study duration. Given the limited representation of older adults, and limited serotype information in our study, we were not able to draw conclusions on patterns of dengue in terms of age and the serotype.

While the patterns with respect to age seem to differ from those reported by Abe et al., it should be noted that, although the study was conducted in the same study area and during an overlapping study period with similar designs, there were differences in study methods (sample collection of just acute serum without a convalescent sample, lab testing in the study by Abe et al.). For example, the study by Abe et al. only used PCR to confirm dengue and there was no other testing applied. However, a negative PCR result does not mean non-dengue [29] and the study may have missed some dengue-positive cases. And these factors, including subject recruitment and case confirmation, could have influences on some of the differences in terms of age as well as proportions and patterns of dengue cases.

### Clinical characteristics between dengue-positive and non-dengue cases

Based on symptoms, we assessed how the clinical presentation of dengue-positive cases differed from non-dengue cases. The final model, with a priori confounders and controlled for treatment type, higher body temperature, and days prior to onset of fever, showed that dengue-positive cases were more likely to present with retro-orbital and abdominal pain. Retro-orbital pain is one of the common symptoms of dengue and it is listed in the 1997 case definition differentiating probable and confirmed DF and dengue hemorrhagic fever (DHF) and dengue shock syndrome (DSS) [30]. Abdominal pain is included in the revised 2009 WHO case definitions [30,31]. Abdominal pain, in addition to being a common gastrointestinal symptom of dengue, has been found to be associated with hospitalized and more severe dengue [32–34].

In the current study, the clinical diagnosis of suspected dengue was found to be very low, only 3.4% among dengue-positive cases. As shown in this study, dengue often presents without typical signs and symptoms, making it difficult to distinguish [35]. Gabon is not known to be dengue-endemic and local physicians do not consider dengue in their differential diagnosis of dengue-like illness, as there is a low index of suspicion in non-endemic regions [36]. Consequently, an epidemic or peak transmission may be missed [36]. Given our data documenting a considerable level of dengue positivity (n = 119/682), clinicians should consider dengue more frequently in febrile patients, especially if they are negative on malaria RDT. Despite compromised sensitivity and specificity, dengue RDTs may be used more readily to support clinical diagnosis [31]. This problem of dengue being largely unrecognized has also been reported in a case (dengue-RDT positive) cluster study conducted in Angola during the outbreak in 2013 where 10% of the subjects showed evidence of recent DENV infection with none diagnosed with dengue [37].

In the current study, we found 116 hospitalized patients, but 92 of them were non-dengue patients with 24 from dengue-positive patients. The frequencies of hospitalization and mean duration of hospital stay between dengue-positive and non-dengue cases were not significantly different. Of 24 hospitalized dengue patients, we had a complete record of admission on 21 of them and 18 patients were hospitalized for 5 days or less. Based on the record of admission: no complication was reported, and hemorrhagic signs were rarely observed. Despite our limited data on indicators of dengue severity, patients in the current study showed to have mild dengue illness.

Our study had limited diagnostic confirmation by PCR, with laboratory diagnosis based largely on serology. In addition to PCR-confirmed cases, cases seroconverting from IgM or IgG negative in acute to IgM or IgG positive in convalescent were defined to be dengue-confirmed. Reported sensitivity of the IgM and IgG ELISA tests used in this study were 85 and 89% [35,38,39]. Reported estimates of specificity were 97% for the IgM and 64% for IgG ELISA tests [35,38,39]. Especially for IgG ELISA, limited specificity may be a concern in dengue diagnosis. However, most of our dengue-confirmed and positive cases were classified based on paired ELISA covering both acute and convalescent phases, not based on single samples in acute phases. By definition, IgM and IgG seroconversion in paired samples of acute serum (from 1–5 days) and convalescent serum (15–21 days thereafter), constitutes confirmed dengue infection [26]. It has been demonstrated that the reported number of days after onset of symptoms, although subjective and based on self-report, can facilitate more accurate interpretation of dengue serology [35]. Our lab results based on paired serology covering the timeframe of acute and convalescent phases of illness further support accuracy of the laboratory diagnosis in this study.

Another possible limitation is cross-reactivity across flaviviruses when using serology [35,38]. Chikungunya was suspected to be another possible co-circulating virus [15]. In addition to the initial testing of 87 samples using the RT-PCR at IVI, we further tested 62 samples using a commercial triplex (DENV, CHIKV, and ZIKV) kit. Eleven were found to be PCR-positive for dengue, but none for CHIKV or ZIKV. Furthermore, our study did not enroll malaria RDT positive patients in the patient screening process, even though some of them could have had co-infection with dengue. However, overall, such concurrent infection is reported to be uncommon [40,41].

Previous outbreaks in Gabon were mainly due to DENV-2 circulation: in 2007 with emergence of DENV-1, and 2010 with the first detected DENV-3 [14]. Our data were consistent with previous data from Gabon in terms of reported circulation of DENV-2 and DENV-3 [12,14]. Although virus strain information from this study is currently limited, there is a plan to conduct sequencing and phylogenetic analysis.

Dengue epidemics occur at 3–5 year intervals in endemic regions [42]. Therefore, the generalizability of the current study is limited by its duration of 20 months and geographical restriction to Lambaréné and its surroundings. Furthermore, one source of bias could be due to the study design, where cases were enrolled only at one study site of ASH and we missed those community residents with relevant symptoms seeking care at other healthcare providers, such as primary health centers and dispensaries. This may further restrict the generalizability of the findings.

Nonetheless, our study held several strengths not found in previous studies. Unlike previous studies focusing mostly on dengue outbreaks and phylogeny of its strains, this study captured the epidemiology during the time of non-epidemic, with a large sample size, allowing for an exploration of the differences between dengue and non-dengue cases.

## Conclusion

Our data provide evidence for a considerable level of transmission of dengue viruses in Lambaréné and its surroundings, Gabon, where dengue was largely understudied. Dengue should be considered more frequently by clinicians among non-malarial febrile patients as a possible disease in circulation in the studied population and more data from additional prospective and longitudinal studies are needed to further define patterns of dengue in Gabon for improved case detection and monitoring of dengue epidemics.

## Supporting information

S1 Table Demographic and other baseline characteristics of the dengue-confirmed, probable, and non-dengue patients with non-localizing febrile illness identified in the health facility-based fever surveillance established in Lambaréné, Gabon in 2015–2016. (DOCX)

S2 Table Univariate logistic analyses showing significant indicators and their odds ratios of dengue positivity in the health facility-based fever surveillance. (DOCX)

S1 File Strobe Checklist. (DOC)
