# Supplementary material for: Epidemiology of dengue fever in Gabon: Results from a health facility-based fever surveillance in Lambaréné and its surroundings
Source: PLoS Negl Trop Dis. 2021 Feb 10;15(2):e0008861. doi: 10.1371/journal.pntd.0008861 (PMC7875424; doi:10.1371/journal.pntd.0008861)
Supplement: S1 Table — (DOCX) [file pntd.0008861.s001.docx]

S1 Table. Demographic and other baseline characteristics of the dengue-confirmed, probable, and non-dengue patients with non-localizing febrile illness identified in the health facility-based fever surveillance established in Lambaréné, Gabon in 2015-2016

| Characteristics | Dengue-confirmed  (n=94) | Dengue-probable (n=25) | Non-dengue  (n=563) | p-value |
| --- | --- | --- | --- | --- |
| Mean age (SD) | 8.68 (6.24) | 10.0 (7.73) | 9.19 (7.88) | 0.714 |
| Age group (years) |  |  |  |  |
| 1-4 | 32 (34.0) | 8 (32.0) | 193 (34.3) | 0.707 |
| 5-9 | 28 (30.0) | 7 (28.0) | 168 (29.8) |  |
| 10-14 | 15 (16.0) | 4 (16.0) | 86 (15.3) |  |
| 15-19 | 11 (11.7) | 2 (8.0) | 70 (12.4) |  |
| 20-24 | 8 (8.5) | 3 (12.0) | 32 (5.7) |  |
| 25-34 | 0 | 1 (4.0) | 3 (0.5) |  |
| 35-44 | 0 | 0 | 5 (0.9) |  |
| 45-55 | 0 | 0 | 6 (1.1) |  |
| Fever duration prior to visit |  |  |  | **0.037** |
| 1-2 days | 25 (26.6) | 4 (16.0) | 197 (35.0) |  |
| 3 days | 39 (41.5) | 7 (28.0) | 182 (32.3) |  |
| 4-7 days | 30 (31.9) | 14 (56.0) | 184 (32.7) |  |
| Fever duration, entire illness | 3.30 (1.17) | 3.84 (1.43) | 3.18 (1.23) | **0.025** |
| Temperature at presentation (SD) | 38.42 (0.92) | 38.30 (0.78) | 38.29 (0.80) | 0.365 |
| Temperature at enrollment |  |  |  | 0.720 |
| Below 38.5°c | 67 (71.3) | 19 (76.0) | 423 (75.1) |  |
| ≥ 38.5°c | 27 (28.7) | 6 (24.0) | 140 (24.9) |  |
| Prev. dengue infection (self-report) | 0 | 0 | 0 | - |
| YF vaccination | 66 (70.2) | 19 (76.0) | 388 (68.9) | 0.740 |
| IPD/OPD | 16 (17.0)/78 (83.0) | 8 (32.0)/17 (68.0) | 92 (16.3)/471 (83.7) | 0.125 |
| Female | 40 (42.6) | 9 (36.0) | 272 (48.3) | 0.309 |
| Clinical diagnosis |  |  |  |  |
| Suspected dengue | 2 (2.1) | 2 (8.0) | 1 (0.2) | **0.001** |
| Non-dengue | 92 (97.9) | 23 (92.0) | 562 (99.8) |  |
| Viral syndrome (% of non-dengue) | 34 (36.2) | 6 (24.0) | 207 (36.8) |  |
| Malaria | 31 (33.0) | 13 (52.0) | 192 (34.2) |  |
| Diarrheal illness | 13 (13.8) | 2 (8.0) | 84 (15.0) |  |
| Bronchitis | 3 (3.2) | 1 (4.0) | 35 (6.2) |  |
| URI | 2 (2.1) | 0 | 11 (2.0) |  |
| Otitis media | 2 (2.1) | 0 | 3 (0.5) |  |
| Other | 7 (7.6) | 1 (4.0) | 30 (5.3) |  |
| Signs and symptoms (presence) |  |  |  |  |
| Rash | 5 (5.3) | 1 (4.0) | 24 (4.3) | 0.845 |
| Fatigue/weakness | 41 (43.6) | 12 (48.0) | 195 (34.6) | 0.115 |
| Headache | 39 (41.5) | 12 (48.0) | 226 (40.1) | 0.724 |
| Retro-orbital pain | 8 (8.5) | 1 (4.0) | 16 (2.8) | **0.025** |
| Neck pain | 1 (1.1) | 1 (4.0) | 2 (0.4) | 0.065 |
| Ear pain | 2 (2.1) | 0 | 5 (0.9) | 0.434 |
| Breathing difficulty | 3 (3.2) | 1 (4.0) | 15 (2.7) | 0.660 |
| Nasal congestion | 19 (20.2) | 5 (20.0) | 130 (23.1) | 0.786 |
| Rhinorrhea | 13 (13.8) | 3 (12.0) | 103 (18.3) | 0.523 |
| Sore Throat | 2 (2.1) | 1 (4.0) | 9 (1.6) | 0.314 |
| Cough | 49 (52.1) | 12 (48.0) | 315 (56.0) | 0.604 |
| Sputum production | 37 (39.4) | 9 (36.0) | 242 (43.0) | 0.655 |
| Nausea & vomiting | 37 (39.4) | 12 (48.0) | 240 (42.6) | 0.709 |
| Diarrhea | 25 (26.6) | 10 (40.0) | 187 (33.2) | 0.323 |
| Constipation | 9 (9.6) | 1 (4.0) | 26 (4.6) | 0.135 |
| Abdominal pain | 32 (34.0) | 11 (44.0) | 144 (25.6) | **0.039** |
| Loss of appetite | 64 (68.1) | 18 (72.0) | 406 (72.1) | 0.724 |
| Muscle pain | 23 (24.5) | 5 (20.0) | 124 (22.0) | 0.837 |
| Joint pain | 23 (24.5) | 5 (20.0) | 126 (22.4) | 0.861 |

≥: greater than or equal to; °C: degree Celsius; IPD: Inpatient Department; OPD: Outpatient Department; SD: Standard Deviation; URI: Upper respiratory infection; YF: Yellow Fever.
