# Supplementary material for: Epidemiology of dengue fever in Gabon: Results from a health facility-based fever surveillance in Lambaréné and its surroundings
Source: PLoS Negl Trop Dis. 2021 Feb 10;15(2):e0008861. doi: 10.1371/journal.pntd.0008861 (PMC7875424; doi:10.1371/journal.pntd.0008861)
Supplement: S2 Table — (DOCX) [file pntd.0008861.s002.docx]

S2 Table. Univariate logistic analyses showing significant indicators and their odds ratios of dengue positivity in the health facility-based fever surveillance

| Characteristics | Total N | N (%) dengue positive  (n=119) | N (%)  Non- dengue  (n=563) | Univariate analysis  Dengue-positive  vs. no dengue | | |
| --- | --- | --- | --- | --- | --- | --- |
|  |  |  |  | OR | 95% CI | p-Value |
| Age group (years) |  |  |  |  |  | 0.957 |
| 1-4 | 233 | 40 (17.2) | 193 (82.8) | Ref | - |  |
| 5-9 | 203 | 35 (17.2) | 168 (82.8) | 1.01 | 0.61-1.66 |  |
| 10-14 | 105 | 19 (18.1) | 86 (81.9) | 1.07 | 0.58-1.95 |  |
| 15-19 | 83 | 13 (15.7) | 70 (84.3) | 0.90 | 0.45-1.77 |  |
| 20-55 | 58 | 12 (20.7) | 46 (79.3) | 1.26 | 0.61-2.59 |  |
| Gender |  |  |  |  |  |  |
| Male | 361 | 70 (19.4) | 291 (80.6) | Ref | - |  |
| Female | 321 | 49 (15.3) | 272 (84.7) | 0.75 | 0.50-1.12 | 0.157 |
| Treatment type |  |  |  |  |  | 0.314 |
| OPD | 566 | 95 (16.8) | 471 (83.2) | Ref | - |  |
| IPD | 116 | 24 (20.7) | 92 (79.3) | 1.29 | 0.78-2.13 |  |
| Fever duration prior to visit |  |  |  |  |  | 0.083 |
| 1-2 days | 226 | 29 (12.8) | 197 (87.2) | Ref | - |  |
| 3 days | 228 | 46 (20.2) | 182 (79.8) | 1.72 | 1.04-2.85 |  |
| 4-7 days | 228 | 44 (19.3) | 184 (80.7) | 1.62 | 0.98-2.71 |  |
| Temperature at enrollment |  |  |  |  |  | 0.514 |
| Below 38.5°c | 509 | 86 (16.9) | 423 (83.1) | Ref | - |  |
| ≥ 38.5°c | 173 | 33 (19.1) | 140 (80.9) | 1.16 | 0.74-1.81 |  |
| YF vaccination ^A^ (*ref.*  not received vaccination) | 473 | 85 (18.0) | 388 (82.0) | 1.13 | 0.73-1.74 | 0.589 |
| Presence of signs and symptoms (*ref.* absence) |  |  |  |  |  |  |
| Rash | 30 | 6 (20.0) | 24 (80.0) | 1.19 | 0.48-2.98 | 0.707 |
| Fatigue* | 248 | 53 (21.4) | 195 (78.6) | **1.52** | **1.02-2.26** | **0.042** |
| Retro-orbital pain* | 25 | 9 (36.0) | 16 (64.0) | **2.80** | **1.21-6.49** | **0.017** |
| Headache | 277 | 51 (18.4) | 226 (81.6) | 1.12 | 0.75-1.67 | 0.584 |
| Nasal congestion | 154 | 24 (15.6) | 130 (84.4) | 0.84 | 0.52-1.37 | 0.489 |
| Rhinorrhea | 119 | 16 (13.5) | 103 (86.6) | 0.69 | 0.39-1.23 | 0.208 |
| Breathing difficulty | 19 | 4 (21.1) | 15 (79.0) | 1.27 | 0.41-3.90 | 0.675 |
| Sore throat | 12 | 3 (25.0) | 9 (75.0) | 1.59 | 0.43-5.98 | 0.489 |
| Cough | 376 | 61 (16.2) | 315 (83.8) | 0.83 | 0.56-1.23 | 0.350 |
| Sputum production | 288 | 46 (16.0) | 242 (84.0) | 0.84 | 0.56-1.25 | 0.386 |
| Nausea & vomiting | 289 | 49 (17.0) | 240 (83.0) | 0.94 | 0.63-1.41 | 0.771 |
| Diarrhea | 222 | 35 (15.8) | 187 (4.2) | 0.84 | 0.54-1.29 | 0.422 |
| Constipation | 36 | 10 (27.8) | 26 (72.2) | 1.90 | 0.89-4.04 | 0.098 |
| Abdominal pain* | 187 | 43 (23.0) | 144 (77.0) | **1.65** | **1.08-2.50** | **0.020** |
| Loss of appetite | 488 | 82 (16.8) | 406 (83.2) | 0.86 | 0.56-1.32 | 0.482 |
| Muscle pain | 152 | 28 (23.5) | 124 (81.6) | 1.09 | 0.68-1.74 | 0.720 |
| Joint pain | 154 | 28 (18.2) | 126 (81.8) | 1.07 | 0.67-1.70 | 0.785 |

Statistical significance of the frequencies: *p-value<0.05 **p-value<.001

^A^based on self-report

≥: greater than or equal to; IPD: Inpatient Department; OPD: Outpatient; YF: Yellow Fever.
